# Inflammation context in Alzheimer’s disease, a relationship intricate to define

**DOI:** 10.1186/s40659-022-00404-3

**Published:** 2022-12-23

**Authors:** Catalina Novoa, Paulina Salazar, Pedro Cisternas, Camila Gherardelli, Roberto Vera-Salazar, Juan M. Zolezzi, Nibaldo C. Inestrosa

**Affiliations:** 1 Centro de Envejecimiento y Regeneración (CARE-UC), Facultad de Ciencias Biológicas, Pontificia Universidad Católica de Chile, Alameda Bernardo O’Higgins 340, P.O. Box 114-D, Santiago, Chile; 2 Centro de Excelencia en Biomedicina de Magallanes (CEBIMA), Escuela de Medicina, Universidad de Magallanes, Punta Arenas, Chile; 3 Instituto de Ciencias de la Salud, Universidad de O’Higgins, Rancagua, Chile; 4 Facultad de Ciencias Médicas, Escuela de Kinesiología, Universidad de Santiago de Chile, Santiago, Chile

## Abstract

Alzheimer’s disease (AD), the most common form of dementia, is characterized by the accumulation of amyloid β (Aβ) and hyperphosphorylated tau protein aggregates. Importantly, Aβ and tau species are able to activate astrocytes and microglia, which release several proinflammatory cytokines, such as tumor necrosis factor α (TNF-α) and interleukin 1β (IL-1β), together with reactive oxygen (ROS) and nitrogen species (RNS), triggering neuroinflammation. However, this inflammatory response has a dual function: it can play a protective role by increasing Aβ degradation and clearance, but it can also contribute to Aβ and tau overproduction and induce neurodegeneration and synaptic loss. Due to the significant role of inflammation in the pathogenesis of AD, several inflammatory mediators have been proposed as AD markers, such as TNF-α, IL-1β, Iba-1, GFAP, NF-κB, TLR2, and MHCII. Importantly, the use of anti-inflammatory drugs such as NSAIDs has emerged as a potential treatment against AD. Moreover, diseases related to systemic or local inflammation, including infections, cerebrovascular accidents, and obesity, have been proposed as risk factors for the development of AD. In the following review, we focus on key inflammatory processes associated with AD pathogenesis.

## Introduction

Neurodegenerative diseases are a common cause of morbidity. In 2011, the worldwide cases per 10,000 inhabitants numbered 400 for Alzheimer’s disease (AD), 315 for Parkinson's disease and 30 for multiple sclerosis [1, 2]. According to the World Health Organization (WHO), approximately 50 million people have dementia, and there are nearly 10 million new cases every year worldwide. AD is the most common form of dementia and may contribute to 60–70% of cases. Most neurodegenerative diseases are related to age, thus the most vulnerable population is elderly individuals, who are more likely to suffer from AD when external factors are present, such as obesity, which exacerbates inflammation [3].

Inflammation is known to exacerbate AD pathogenesis [4]. Therefore, several research groups have proposed to treat AD with anti-inflammatory drugs. In fact, nonsteroidal anti-inflammatory drugs (NSAIDs) have been shown to delay dementia in AD [5]. NSAIDs are some of the most widely used drugs in developed countries, and their consumption continues to grow every year. In the United States, the use of NSAIDs increased by 125% from 1999 to 2002. In Spain, it was estimated that more than four million people used NSAIDs in 1999, of which 30–40% were older than 65 years. In 2005, undeveloped countries such as Costa Rica spent two million dollars on NSAIDs, which represented an increase of 25% from the expenditure in 2001 [6]. Despite advances in drugs, inflammation has not been eradicated in neurodegenerative diseases, and research on how to counteract its effects is ongoing. Numerous studies using different animal models have resulted in vital discoveries about inflammation and neuroinflammation. For example, our lab has shown that administration of andrographolide (ANDRO), a natural compound, decreases neuroinflammation in the rodent *Octodon degus,* which develops AD spontaneously [7]. Another study using an AD mice model induced by Aβ_1–42_ injection has demonstrated that ginsenoside Rb1 (GsRb1) reduces neuroinflammation in the hippocampus [8]. Additional studies have revealed a potential mechanism of action of anti-Aβ antibodies via experiments on transgenic mice overexpressing mutant human amyloid precursor protein (PDAPP) under the control of the mini-promoter of platelet-derived growth factor (PDGF) [9].

In this review, we focus mainly on the relationship between inflammation and AD through topics ranging from neurodegeneration to the roles of highly insoluble Aβ deposits and neurofibrillary tangles as inflammatory stimuli in the brains of AD patients [10]. These topics are of particular interest due to the effects of misfolded and aggregated proteins; when these proteins bind to pattern recognition receptors in microglia and astroglia, they trigger an innate immune response characterized by the release of inflammatory mediators. Accumulation of these mediators culminates in chronic neuroinflammation that exacerbates AD pathogenesis [4, 10]. External factors, including systemic inflammation, such as that observed in obesity, are likely to interfere with the brain's immune processes and further promote disease progression. Modulation of risk factors and targeting of these immune mechanisms could lead to future therapeutic or preventive strategies for AD [4].

## Inflammation and neuroinflammation

To elucidate the relationship between inflammation and AD, it is necessary to understand the basics of inflammation, discuss the connection to age-related diseases, and then describe how neuroinflammation works. Inflammation is a response of the immune system to infections, tissue damage, or other harmful conditions [11, 12]. At the cellular level, the cells responsible for inflammation are immune cells such as macrophages, dendritic cells, mast cells, neutrophils, and lymphocytes, and nonimmune cells such as epithelial cells, endothelial cells, and fibroblasts. The molecules responsible for inflammation can be generally divided into cytokines and transcription factors [13].

It is important to differentiate acute inflammation from chronic inflammation. Acute inflammation is the first reaction of innate immunity and corresponds to an adaptive response, which restores cell and tissue homeostasis. The inflammatory response typically ends once the thread of damage stops, for example, when tissue repair is prepared to commence or when the pathogen is removed. This process is called the resolution of inflammation and is mediated by several anti-inflammatory mediators, such as interleukin 10 (IL-10), transforming growth factor beta (TGF-β), and glucocorticoids [13]. If this process fails, the consequences are harmful and can affect several physiological functions, such as cardiovascular, respiratory, and neuronal [13–15]. In this case, acute inflammation transforms into chronic inflammation. The inflammatory pathways vary with the stimulus, and the duration of the inflammatory responses changes with the level of damage. However, in most cases, these responses cause systemic effects due to the excessive production of inflammatory cytokines, leading to chronic inflammation. Chronic inflammation is the key driver of pathogenesis in several diseases, and the main damage caused to the host is mediated by the host's own inflammatory response rather than by pathogens [13]. Today, chronic inflammation is known to play a role in many diseases, such as atherosclerosis, obesity, type 2 diabetes, asthma, inflammatory bowel diseases, rheumatoid arthritis, cancer and neurodegenerative diseases [3].

Most age-related diseases, such as cardiovascular disease, cancer, metabolic syndrome, osteoporosis, dementia and AD, have an inflammatory pathogenesis [16]. In neurodegenerative diseases, inflammation has its own unique process that may present in two ways. In peripheral inflammation, cytokines such as TNF-α, interleukin 6 (IL-6) and interleukin 1β (IL-1β) can affect and cross the blood–brain barrier (BBB), stimulating it to release proinflammatory mediators and making it more permeable to cells, thus allowing the passage of leukocytes to the brain [17]. These phenomena cause a cascade of events within the brain leading to microglial and astroglial responses involving further production of proinflammatory mediators, ROS, RNS, among others. This mechanism is defined as neuroinflammation [18]. Neuroinflammation can occur, as mentioned, or can be triggered by the brain's own immune response, which depends on the glial cells. Neuronal lesions or highly insoluble proteins, such as Aβ, can activate these cells without leukocyte infiltration [19]. In both cases, neuroinflammation can culminate in synaptic deterioration, neuronal death, and exacerbation of various brain pathologies [20]. It is also important to mention that at the cellular level, astrocytes, microglia, neurons, and infiltrated leukocytes are responsible for neuroinflammation [18].

## CNS cells affected by neuroinflammation in AD

The brain's innate immune system is made up mainly of microglia and astrocytes, both types of glial cells [21]. Microglia, the only immune-derived cells within the brain, are responsible for immune surveillance and response processes, including phagocytosis. On the other hand, astrocytes fulfill several homeostatic functions in the central nervous system (CNS), regulate immune responses by releasing cytokines, and act as antigen-presenting cells [22, 23]. During the chronic phase of neuroinflammation and early AD pathogenesis, the activation of microglia and astrocytes appears to play a positive effect, contributing to Aβ elimination. However, as the disease progresses, continuous activation of microglia and astrocytes in the brain can promote AD pathology [19, 24]. Indeed, sustained glial responses have been shown to correlate with Aβ plaque burden, dystrophic neurite growth, and phosphorylation of tau [25, 26].

### Microglia as CNS cells affected by neuroinflammation in AD

In healthy brains, microglia are in an inactive or resting state and are distinguished both by their branched morphology and by their low expression of major histocompatibility complex (MHC) proteins and other antigen-presenting surface receptors [27, 28]. However, when extracellular signals, such as pathogens, foreign materials (such as Aβ), or dead cells, are present in the CNS, microglia undergo a morphological change to an amoeboid form, regarded as “activated” microglia [24]. As a consequence, cytokines and proinflammatory mediators, such as IL-1β, IL-6, and TNF-α, and ROS are released, leading to neuronal damage [19]. This uncontrolled and sustained activation of the microglia state is described as reactive gliosis and has been implicated in AD pathogenesis [29]. Interestingly, several studies have shown that activated microglia are predominantly found in close vicinity to Aβ plaques [30–32]. Similarly, the formation and appearance of Aβ plaques in the brain correlate with the activation of microglia [33]. As a consequence, this microglia accumulation can worsen AD pathology and neurodegenerative processes by overexpressing proinflammatory cytokines, which in turn may lead to a reduction in Aβ clearance and accumulation in the brain [24]. For example, interferon γ (IFNγ) and TNF-α, two chemokines released by activated microglia, have been demonstrated to decrease Aβ degradation and increase its production [34, 35]. Contrarily, genetic deletion of IFNγ receptor was shown to reduce microglial activation and Aβ generation, together with the prevention of cognitive decline [34, 36].

### Astrocytes as CNS cells affected by neuroinflammation in AD

Astrocytes are CNS resident cells and play several key roles in brain homeostasis maintenance, including neurotransmitter uptake, blood flow regulation, and support of neuronal metabolism, among others [37]. However, as a response to many pathological situations, including trauma, neuroinflammation, or neurodegeneration, these homeostatic astrocytes change to a reactive phenotype and induce astrogliosis [38]. The latter is described as an abnormal increase of astrocytes, and it is characterized by cellular hypertrophy and an increase in the glial fibrillary acidic protein (GFAP) expression [39]. Moreover, activated astrocytes exert neurotoxic effects with loss of neurotrophic functions, causing a chain reaction. These events are associated with increased release of cytokines and inflammatory mediators, neurodegeneration, decreased uptake of glutamate, and loss of neuronal synapses [24]. In AD, astrogliosis can be triggered both by damaged neurons or glia and by extracellular deposits of Aβ [40–42]. Indeed, Aβ can induce signals and spontaneous oscillations of intracellular calcium concentrations in astrocytes that are partially responsible for neurotoxicity [43]. In addition, studies in cultures have shown that Aβ decreases the expression and capacity of two astrocytic glutamate transporters, glutamate-aspartate transporters, and glutamate transporter 1, which culminates in a decrease in glutamate uptake by astrocytes, inducing excitotoxicity [43, 44]. Another characteristic of astrocytes that defines them as detrimental in the pathogenesis of AD is their release of proinflammatory cytokines and ROS, which may also lead to neuroinflammation and neurotoxicity [45].

## Neuroinflammation related to Aβ and tau

The presence of aggregated Aβ and tau are hallmark pathogenic features in AD and can be found in the early and late stages of the disease. Importantly, inflammation can trigger Aβ or tau overproduction, which in turn induces inflammatory responses, resulting in a vicious cycle of neuroinflammation and pathology [46–48].

### Neuroinflammation related to Aβ

**Fig. 1:**
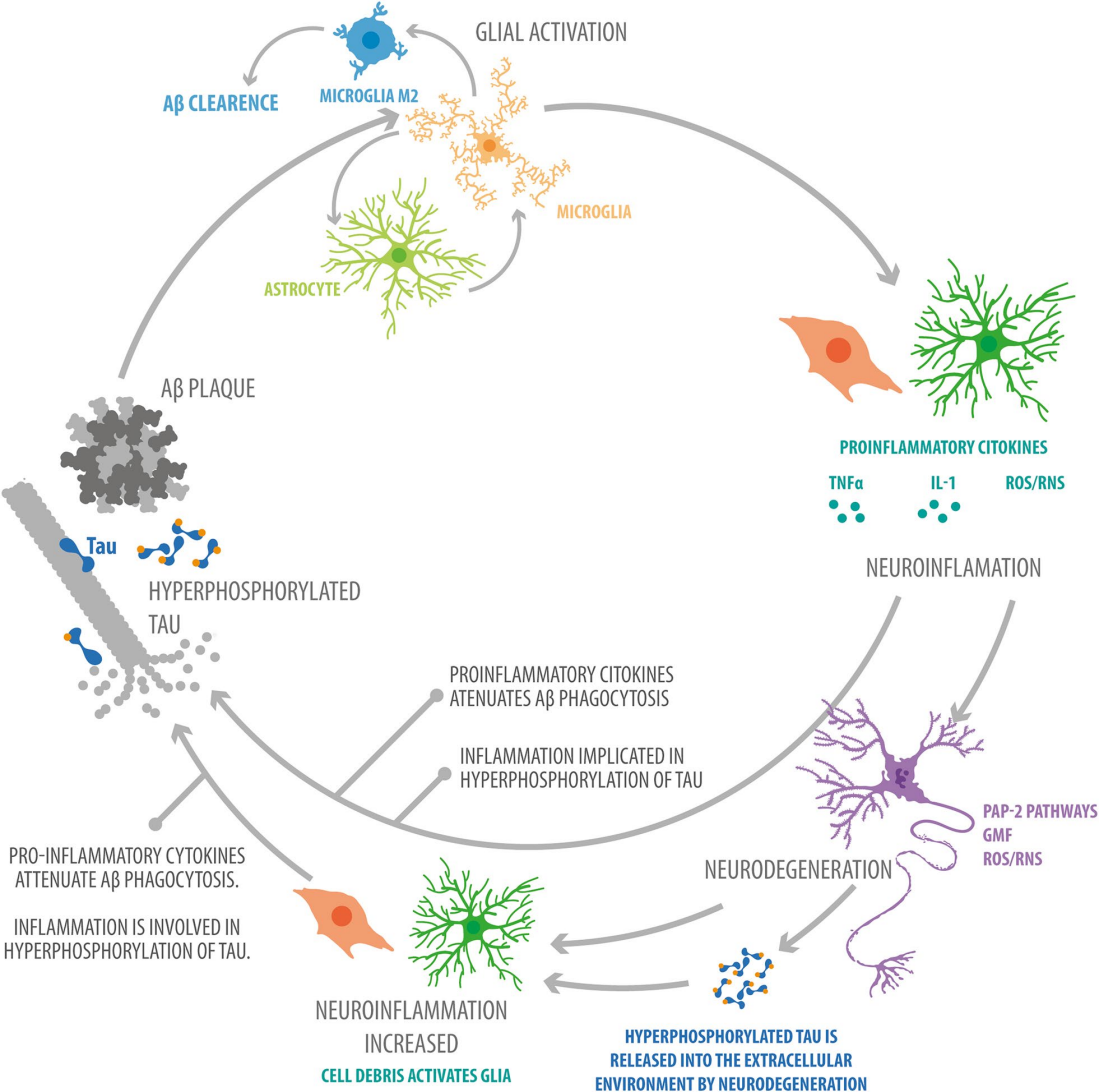
Microglial M2 and M1 phenotypes during AD. Two microglial activation phenotypes fluctuate in AD: M1, the classic and inflammatory phenotype (associated with release of ROS, RNS, and proinflammatory cytokines such as TNF-α and IL-1β); and M2, the alternative and anti-inflammatory phenotype (associated with release of anti-inflammatory cytokines such as IL10 and IGF1), which is responsible for Aβ phagocytosis. M1-to-M2 polarization is stimulated by proinflammatory cytokines such as IL4 and IL13, while M2-to-M1 polarization is stimulated by a lack of anti-inflammatory cytokines such as IL10 and IGF1. The M1 phenotype is seen in earlier stages of the disease, while the M2 phenotype is seen later. An example of a receptor involved in M2 activation is the nuclear receptor PPARγ, and an example of a pathway involved in M2 activation is the LKB1-AMPK pathway. An example of a receptor involved in M2 activation is the TLR2 receptor, which is capable of binding to Aβ, and an example of a pathway involved in M2 activation is the MYD88 pathway

The appearance of amyloid plaques, which are primarily composed of Aβ, is accompanied by strong activation of microglia in the vicinity of the plaques, suggesting that Aβ acts as one of the primary drivers of microglial activation. Indeed, injecting Aβ directly into the brain has been shown to induce microglial activation and neuronal loss [49]. However, studies using transgenic mice overexpressing amyloid precursor protein (APP) have revealed contradictory effects on AD pathogenesis depending on the microglial phenotype [50]. For example, M1 microglia (classically activated) produce neurotoxic proinflammatory mediators, including cytokines (e.g., TNF-α and IL-1β), chemokines (e.g., CCL2), and ROS/RNS. Overall, this neuroinflammatory response leads to neuronal damage. In addition, the microglial clearance mechanisms of Aβ participate in localized inflammatory mechanisms that can be cytotoxic to nearby tissue [51]. On the other hand, during M2 microglia phenotype (alternative macrophage activation), phagocytosis of Aβ occurs, and anti-inflammatory and neurotrophic mediators are expressed and produced, such as arginase 1, BDNF, IGF-1, and IL-10. These molecules provide protection to neurons and have been associated with neuronal regeneration (Fig. 1) [52]. It is important to mention that the microglial activation phenotype may change during AD. In the early stage of the disease, M2 microglia predominate to engulf the Aβ and produce anti-inflammatory factors to quench proinflammation and maintain tissue homeostasis. However, the persistent presence of Aβ plaques promotes polarization of microglia toward the M1 phenotype, thus compromising the immune resolution process in the later stage of disease progression and leading to neuronal degeneration (Fig. 1) [53].

**Fig. 2:**
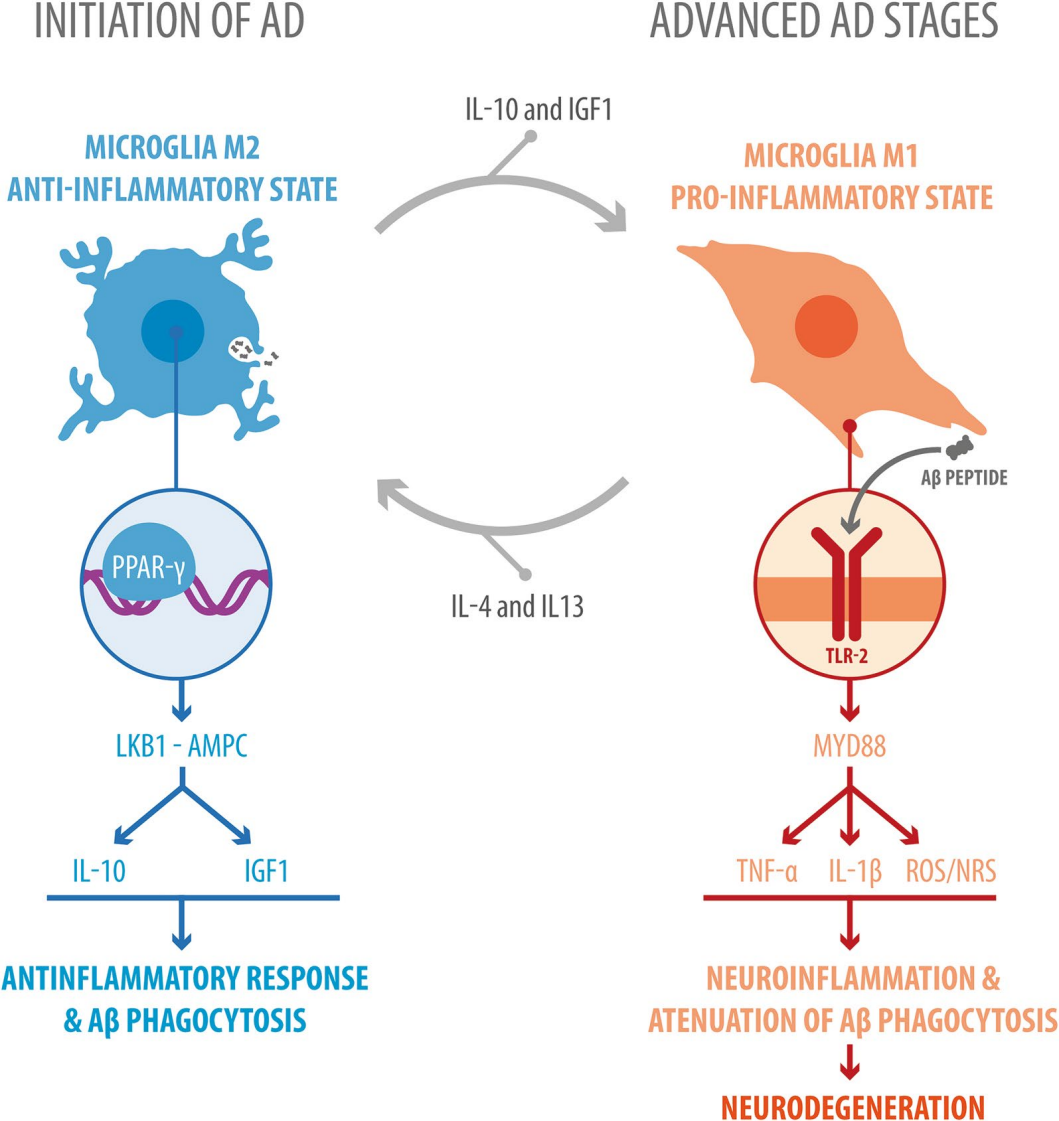
Vicious cycle of neuroinflammation with Aβ and tau. Aβ and tau activate microglia and astrocytes to produce ROS, RNS, and proinflammatory cytokines such as IL-1 and TNF-α, inducing neuroinflammation. In turn, neuroinflammation increases the levels of Aβ and tau (because it is involved in the hyperphosphorylation of tau and attenuates the clearance of Aβ) or causes neurodegeneration in different ways (for example, via the PAP-2, GMF, ROS and RNS pathways). Neurodegeneration exacerbates neuroinflammation because the remains of dead cells and the tau that remains in the extracellular environment due to cell death activate microglia and astrocytes. This increased neuroinflammation also increases the Aβ and tau levels (for the same reasons as in the initial neuroinflammation)

Due to the action of Toll-like receptors (TLRs) present in microglia, Aβ ultimately causes neuroinflammation [54]. For example, one study found that TLR2 interacts directly with Aβ42 [52]. In fact, when TLR2 is expressed in HEK293 cells that do not express TLR2 endogenously, the cells respond to Aβ42 and release IL-8 (a proinflammatory cytokine). The leucine-rich repeats (LRRs) in the N-terminal domain of the TLR2 receptor are partly responsible for this ligand-receptor interaction, but not completely, because TLR1 and TLR3 also contain LRRs and do not bind to Aβ. Another important finding from the same study was the identification, by directed mutagenesis, of the amino acids EKKA (741–744) as a critical cytoplasmic domain for inflammatory signal transduction. The results also showed that the TLR-MyD88 signaling pathway controls M1 microglial activation. In fact, the authors observed that myeloid differentiation primary response 88 (MyD88) deficiency increases Aβ phagocytosis but decreases inflammatory activation. These findings suggest that the signaling pathway that controls M1 microglial activation in response to Aβ is separated from the signaling pathway that activates M2 microglia in response to Aβ and increases phagocytosis of Aβ. Furthermore, the authors observed that in transgenic mouse-derived bone marrow (BM) precursors with chimeric AD amyloid, that is, TLR2-deficient BM-derived microglia, more M2 microglia were activated than M1 microglia, which reduces neuroinflammation. In summary, Aβ binds to TLR2, causing M1 microglial activation, and signal transduction through the MyD88 pathway ends with the release of proinflammatory mediators that induce neuroinflammation. This process contributes to the dysfunction and neuronal loss characteristic of the pathogenesis of AD (Fig. 2) [50, 52].

Efforts have been made to study the relationship among signaling pathways, microglia, and AD. Indeed, recent evidence has shown that adenosine monophosphate-activated kinase (AMPK) signaling is involved in microglial polarization [55]. Also, peroxisome proliferator-activated receptor γ (PPARγ) has been shown to regulate the M2 microglial activation (Fig. 2). In fact, in one study, increased mRNA levels of the M2 marker YM1 were observed when cells were treated with PPARγ agonists [56]. In addition, several studies have revealed that treatment with agonists of PPARγ reduces Aβ levels in the CNS and alleviates AD pathology [56–58]. The modulating effect of PPARγ on microglial polarization could be due to activation of the LKB1-AMPK signaling pathway; this is inferred because in one experiment, the results indicated that treatment with the LKB1 inhibitor radicicol or removal of LKB1 prevented the activation of AMPK signaling and the phenotypic shift from M1 to M2 in the BV2 microglial cell line treated with LPS. This information could be used for future AD treatment studies [59, 60].

Although gliosis has been mostly shown to occur as a consequence of amyloid plaques, early evidence suggests that Aβ and tau oligomeric species can also activate the immune system. Indeed, several studies have shown the activation of different microglial and astrocytic markers and the release of proinflammatory molecules prior to the deposition of Aβ plaques [61–64]. More importantly, the inflammatory response appears to correlate with the concentration of oligomers but not to the plaque burden or monomeric Aβ. Though it is not yet entirely understood how these toxic oligomers perform their activity [63], they have been associated with several microglial and astrocytic membrane proteins. Many of these receptors, including N-methyl-D-aspartate (NMDA) and α-amino-3-hydroxy-5-methyl-4-isoxazolepropionic acid (AMPA) receptors, cause a fast influx of Ca^2+^, triggering inflammation[65–67]. In addition, Aβ can also bind the receptor for advanced glycation end products (RAGE), a surface molecule found in microglia, astrocytes, and cerebral endothelial cells, which mediates immune responses [68]. Interestingly, Aβ oligomers have been shown to significantly upregulate RAGE levels [69–71], however, it remains controversial which Aβ species induces inflammation[72].

### Neuroinflammation related to tau

In addition to the presence of Aβ plaques, another characteristic pathogenic feature of AD is the presence of intraneuronal neurofibrillary tangles (NFTs) that consist of aggregated and abnormally hyperphosphorylated tau protein [73]. What triggers the formation of paired helical filaments is currently unknown, however, neuroinflammation could play a role since it is involved in hyperphosphorylation of tau and the formation of NFTs [74]. In turn, the presence of NFT exacerbate neuroinflammation and neurodegeneration by binding to microglia and activating them to produce neuroinflammatory mediators [75]. In fact, in a study using P301S-mutant human tau-transgenic mice, it was observed that microglial activation preceded the formation of tangles, and immunosuppression in these mice decreased tau pathology and increased life expectancy [74].

The hyperphosphorylated tau protein ultimately causes neurodegeneration in different ways ranging from microtubule destabilization to interaction with the 20S subunit of the proteasome and inhibition of its activity, which contributes to abnormal accumulation of proteins and initiates a cascade of events that leads to neuronal death [75, 76]. Once neuronal death has occurred, hyperphosphorylated tau aggregates are released into the extracellular environment, activating glia, which may lead to neuroinflammation [76]. Although different transgenic models of tauopathy have been used, age-dependent microglial activation-related astrogliosis and neuroinflammation have been observed at a stage in which neuronal loss does not occur [75]. It has been shown that treatment with extracellular tau activates p38 MAPK and alters the proinflammatory cytokine expression pathway in microglia, increasing the gene expression of IL-6, IL-1β, TNF-α and Mip-1α [77]. In addition, microglia activation has been shown to occur through the chemokine receptor CX3CR1 and its ligand fractalkine (CX3CL1) [78]. Under physiological conditions, CX3CR1/ CX3CL1 signaling is involved in the synthesis of anti-inflammatory signals, however, in AD, the expression of CX3CL1 was shown to be decreased. Importantly, tau has been shown to compete with CX3CL1 by binding and triggering the internalization of CX3CR1 [79], which can accelerate tau pathology and memory impairment [73, 80].

Similar to Aβ, tau oligomers have also been shown to initiate an inflammatory response [81]. Recent evidence demonstrated that tau oligomers promote the release of high mobility group box 1 (HMGB1), a highly expressed protein in AD which is able to activate RAGE and TLR4, promoting the expression of inflammatory cytokines [81, 82]. Additional studies also found a tau oligomer-dependent activation of microglia; however, the mechanism remains less clear [83, 84]. The effects of both Aβ accumulation and tau hyperphosphorylation on glial activation and neuroinflammation are presented in Fig. 2.

## Inflammation markers in AD

Inflammatory markers have begun to be exploited for age-related diseases. Among the inflammatory markers whose levels are increased in age-related diseases are IL-1β and TNF-α. Moreover, the transcription factors that regulate chronic inflammation in different diseases are nuclear factor kappa-activated light chain B (NF-κB), signal transducer and activator of transcription (STAT). These proteins positively regulate several genes that encode proinflammatory cytokines and can also be used as inflammatory markers [3]. Inflammatory markers are crucial in the study and diagnosis of AD. As stated earlier, inflammation participates in the development of AD and is present in CNS cells, such as microglia and astrocytes; as well as in pro-inflammatory mediators and transcription factors (Table 1).

**Table 1:** Descriptions of different inflammatory markers used for the study or diagnosis of AD

Marker	Species in which it exists related with AD studies	Potential use in clinical diagnosis	Function in inflammation	References
TLR2	Human, mouse, rat	- Non-alcoholic fatty liver disease (NAFLD) - Non-alcoholic steatohepatitis (NASH) - Chronic lymphocytic leukemia - Acute coronary syndrome	Receptor which interacts with Aβ	[52, 194–199]
MHCII	Human, mouse, rat	- Triple-negative breast cancer (TNBC) - Systemic lupus erythematosus - Ovarian cancer	Antigen presenter	[200–205]
Iba-1	Human, mouse, rat	- Traumatic brain injury - Frontotemporal lobar degeneration - Epilepsy - Postoperative cognitive decline	Intracellular protein of microglia, related to support the phagocytosis process	[206–212]
GFAP	Human, mouse, rat	- GFAP autoimmunity - Traumatic brain injury - Alexander disease - Multiple sclerosis	Mediates astroglia cell activation	[213–220]
TNF-α	Human, mouse, rat	- Psoriasis - Rheumatoid arthritis - Alzheimer’s disease - Type 2 diabetes mellitus	Pro-inflammatory cytokine	[24, 128, 131, 137, 221–225]
IL-1	Human, mouse, rat	- Systemic juvenile idiopathic arthritis - Rheumatoid arthritis - Gout - Familial Mediterranean fever -Muckle-Wells syndrome - Acute myocardial infarction - Heart failure - Type 2 diabetes	Pro-inflammatory cytokine	[24, 225–230]
NF- κB	Human, mouse, rat	- Preeclampsia - Degenerative Disc Disease - Colorectal cancer - Non-medullary thyroid cancer	Transcription factor that activates genes related to inflammation	[133, 231–237]

### Microglial inflammation markers in AD

As stated earlier, microglial activation can be used as an indicator of neuroinflammation in AD, since microglia, in the presence of insoluble proteins such as those found in AD, release proinflammatory factors [35]. For this reason, microglial activation markers can be used as neuroinflammatory markers in AD. Many microglial proteins are used as markers of neuroinflammation, such as TLR2, which interacts with Aβ; MHC II, a protein present in activated microglia that presents digested and phagocytosed fragments of pathogens, increasing the inflammatory response in surrounding microglial cells; and IBA-1, an intracellular calcium-binding protein related to the reorganization and support of the phagocytosis process because of its ability to bind to actin molecules [74, 76, 85].

In addition, genetic factors such as rare variants of the triggering receptor expressed on myeloid cells 2 (Trem2) have been recently suggested to play a crucial role in AD pathogenesis [86]. Trem2 is a type I transmembrane protein which recognizes phospholipids, apoptotic cells and lipoproteins, among others [87–90]. Studies in AD mouse models have suggested that Trem2-dependent activation of microglia is necessary to limit Aβ pathology [91]. Indeed, Trem2 is one of the most highly expressed receptors in microglia and can affect Aβ clearance in a manner mediated by phagocytosis of apoptotic and inflammatory neurons [90, 92, 93]. Interestingly, TREM2 knockout (KO) animals have shown attenuated cytokine production in response to Aβ [94, 95]. Although it is not clear how variants of the TREM2 gene confer a two- to fourfold increased risk for sporadic AD [96, 97], it has been postulated that Trem2 deficiency produces numerous effects. For example, it reduces the viability and proliferation of primary microglia, as microgliosis is reduced in Trem2-/- mouse brains; induces cell cycle arrest at the G1/S checkpoint; and ultimately reduces the stability of catenin, a key component of the canonical Wnt signaling pathway that is responsible for (among many other biological processes) cell survival. Thus, it has been shown that the Trem2-mediated Wnt/β-catenin pathway plays a pivotal role in microglial viability, suggesting that therapeutic modulation of this pathway can help combat the deterioration of microglial survival and microgliosis associated with AD [98]. Moreover, mutations in the TREM2 gene, such as Y38C, have been related to an increased risk of AD comparable to the risk associated with mutations in the allele of the apolipoprotein E (APOE) [96, 99, 100]. However, the effect of Trem2 on Aβ pathology has been inconsistent. Indeed, some studies using Trem2-/- mice have found no change in total plaque load [101, 102], while other studies have found an increase [89, 103] or even a decrease in load [103, 104]. Some of these differences, which may be attributable to the data collection time windows [103], could suggest that the elimination of Aβ is not the only function of Trem2 [105].

Similarly, Trem2 deficiency has been shown to both mitigate neuroinflammation and protect against brain atrophy in the context of tau pathology, as well as to accelerate tau aggregation [106–108]. For example, attenuation of neurodegeneration and significantly reduced microgliosis have been observed in Trem2-deficient mice crossed with the PS19 human tau transgenic line [108]. However, the authors did not see changes in tau phosphorylation or insoluble tau levels. These observations suggest that Trem2 can facilitate a microglial response in the context of tau pathology or tau-mediated damage in the brain without altering tau levels. Furthermore, there is evidence suggesting that microglia may contribute to the neurodegenerative process associated with tau pathology without altering tau aggregation [109]. On the other hand, using a humanized tau model, Bemiller and colleagues showed that Trem2 deficiency altered the microglial response and accelerated the aggregation and hyperphosphorylation of tau [106]. Likewise, the absence of Trem2 has been associated with a decrease in microgliosis in a variety of disease models, but the ultimate effect on the different pathologies and neuronal integrity differs, suggesting that Trem2 mediates microglial responses to amyloidosis even though it does not necessarily affect the total plaque load or protein aggregation [110, 111]. Together, these results suggest dual roles of Trem2 and microglia in the context of both amyloid and tau pathology.

Trem2 signals through different pathways, which can be modulated to antagonize the detrimental effects. For example, recent evidence shows that Trem2 promotes microglial survival by activating the Wnt/β-catenin signaling pathway; thus, it is possible to restore Wnt/β-catenin signaling when Trem2 activity is disrupted or reduced [98]. Similarly, studies using transgenic mouse models of AD indicate that inefficient mTOR signaling in Trem2-deficient microglia is associated with a compensatory increase in autophagy in vitro in AD [112–114]. Likewise, cell membrane phospholipids and lipoprotein particles can continuously activate Trem2, inducing mTOR signaling through upstream activators such as PI3-K, PDK1, and Akt, which are recruited by signaling associated with the Trem2 subunits DNAX-activating protein (DAP12) and DAP10 [114]. Additionally, other groups have reported that Trem2 deficiency alters the expression and secretion of cytokines induced by Aβ, and its downstream signaling alters the expression of proinflammatory cytokines such as IL-6 and macrophage inflammatory protein 1α (MIP-1α, also known as CCL3), and decreases the expression of the anti-inflammatory protein Arg1 [95, 106].

In addition to Trem2, several immune genes have also been implicated in AD pathology. Among them is CD33, a microglial receptor that has been shown to be elevated in AD [115, 116]. Importantly, several polymorphisms in CD33 have been associated with AD susceptibility and a correlation between CD33 expression and cognitive function has been described previously. More importantly, CD33 depletion results in a reduction of Aβ levels, together with a decrease plaque burden [117]. Another immune gene associated with AD is phospholipase C-gamma 2 (PLCG2), which is expressed primarily by microglia and granule cells [118]. Interestingly, PLCG2 expression was also shown to correlate with Aβ density [119]. A third gene recently discovered by genome-wide association studies associated with microglia and AD is inositol polyphosphate-5-phosphatase (INPP5D) [120]. Similar to CD33 and PLCG2, the expression of INPP5D was shown to correlate with AD progression and amyloid plaque density [121].

### Astrocytic inflammation markers in AD

Likewise, astrocyte markers can also be used as indicators of neuroinflammation [122, 123]. Increased GFAP mRNA levels have been associated with AD since GFAP levels in AD brains are almost twice of those in control brains [39]. Furthermore, GFAP levels are strongly and positively correlated with the duration of the disease, suggesting that GFAP can be a useful neuroinflammatory marker in AD [124].

### Proinflammatory cytokines as inflammation markers in AD

Other inflammatory markers in AD are proinflammatory cytokines, such as TNF-α and IL-1 [125]. Indeed, in an early study, Tan and colleagues followed cognitively healthy participants with the aim to determine whether cytokines, such as IL-1, IL-6 and TNF-α levels were associated with AD risks [126]. Interestingly, individuals with the highest production of these cytokines were at higher risk of developing AD. The inflammatory cytokine TNF-α is particularly important in the development of AD since it participates in the spread of inflammation and plays a critical role in the pathophysiology of AD [127]. Several studies have shown that TNF-α levels are significantly higher in patients with AD than in healthy people [128–130]. In fact, in one study, injecting mice with Aβ_1–40_ increased the expression of TNF-α, and worsened cognitive function [131]. Importantly, the cognitive impairment was alleviated by treatment with anti-TNF-α antibodies and led to a reduction of biochemical alterations produced by Aβ_1–40_.

### NF-κB, a transcription factor related to AD

The last marker of inflammation that will be discussed is the transcription factor NF-κB, which regulates the transcription of several proinflammatory genes and is activated by Aβ, tau, ROS, and several cytokines, among other molecules [132, 133].

NF-κB dysregulation has been widely associated with AD and can result in glial cell activation [134]. In addition, a strong activation of NF-κB has been found in cells treated with different Aβ fragments, such as Aβ_1-40_ [133]. In a different study, the activity of the transcription factor NF-κB was measured using a microglial cell line treated with sAPP (the peptide remaining after cleavage of APP with alpha or beta-secretase). Interestingly, sAPP-treated cells responded with the activation of the transcription factor NF-κB, suggesting a close association between these molecules [135]. Indeed, the levels of NF-κB p65, one of the members of the NF-κB family, were found to be significantly increased in the brains of patients with AD, and two functional NF-κB binding elements were identified in the promoter region of the human BACE1 gene [136], which can further worsen AD pathology. Thus, given the association between TNF-κB and neurodegeneration/inflammation, attenuating this pathway could provide a therapeutic avenue to attenuate AD pathology [136, 137].

Similar to Aβ, a recent study found that tau caused the activation of the NF-κB pathway in microglia, which in turn led to an increase in tau seeding and spreading [138]. More importantly, the inactivation of microglial NF-κB was able to restore cognitive deficits and helped to reestablish a homeostatic phenotype in microglia.

## Implications of neuroinflammation in AD

AD is largely characterized by cognitive deficits that arise due to neurodegeneration or synaptic loss [139]. In this section, we will discuss how the neuroinflammation present in AD leads to neurodegeneration and synaptic loss [140].

### Neurodegeneration as an implication of neuroinflammation in AD

Neurodegeneration is a phenomenon that occurs in the CNS through signals associated with the loss of neuronal structure and function [141]. In AD, neurons in the hippocampus and entorhinal cortex are the first to degenerate [142]. Neurodegeneration is mediated, among other factors, by inflammatory and neurotoxic mediators such as IL-1β, TNF-α, ROS, and RNS [143]. These mediators directly or indirectly affect neuronal survival and induce neurodegeneration through glial cells and inflammatory cells. Activated microglia, astrocytes, neurons, T cells, and mast cells release these inflammatory mediators [141]. For example, when there is dysfunction in the BBB caused by microglia-secreted proinflammatory mediators in AD, peripheral inflammatory mediators and immune and inflammatory cells, such as T cells and mast cells, cross the BBB [144]. These mediators and cells activate microglia, astrocytes, and neurons to release even more proinflammatory mediators, increasing the inflammation-mediated neurodegeneration [141]. Several mediators, receptor proteins, or pathways are upregulated or activated in neuroinflammation and mediate neurodegeneration, such as protease-activated receptor 2 (PAR-2), which is expressed in mast cells, glial cells, and neurons. Mast cell activation induces the release of specific types of extracellular vesicles (EVs) with specific inflammatory mediators, including proteases such as tryptase, which activate neurons and glial cells and increase neuroinflammation mediated by the PAR-2 pathway, regulating neurodegeneration [143]. Another mediator is glial maturation factor (GMF), a protein that activates microglia and neurons, causing these cells to release proinflammatory cytokines and thus mediating neurodegeneration [143].

The last examples of neurodegeneration mediators that will be discussed in this section are ROS and RNS. Notably, microglia respond to neuroinflammation by changing their gene expression, including via de novo expression of the inducible isoform of nitric oxide synthase (iNOS), thus triggering oxidative and nitrosative stress [145]. Mitochondria are targets of oxidative and nitrosative stress, as ROS and RNS damage proteins, nucleic acids, polysaccharides, and lipids, which can lead to damage and even cause mutations in mitochondrial DNA. Overall, mitochondrial alterations can result in neurodegeneration [146]. Given the relationship between mitochondrial dysfunction and AD, treatments that target mitochondria, such as the mitochondrial antioxidant Szeto-Schiller peptides, have been widely studied [147]. These peptides have a sequence motif that allows them to target mitochondria, and their antioxidant action can be attributed to tyrosine or dimethyltyrosine (Dmt), which plays a role in mitochondrial ROS clearance. These drugs are currently undergoing phase II clinical trials for the treatment of diseases involving mitochondrial oxidative damage, such as AD and other neurodegenerative diseases [148].

### Synaptic loss as an implication of neuroinflammation in AD

It has been established that neuroinflammation can lead to synaptic loss. Pro-inflammatory cytokines such as IL-1β and TNF-α regulate the transcription of many genes, including genes encoding enzymes in the cascade of arachidonic acid (a precursor of prostaglandin) in various cell types [149]. In the brain, upregulated arachidonic acid and its metabolites influence signal transduction and transcription. For example, they can influence the transcription of synaptic proteins, causing synaptic loss [150]. An example of an altered synaptic protein is the synaptic marker drebrin, whose levels are decreased in AD [151]. In fact, in one experiment, drebrin levels in the superior temporal cortex were found to be approximately 35% lower in subjects with mild cognitive impairment, who exhibited upregulation of TNF-α and IL-6, than in subjects without cognitive impairment [152].

## Anti-inflammatory drugs for AD treatment

NSAIDs are widely used medications to reduce pain, fever, and other inflammatory processes and exert their anti-inflammatory function by inhibiting the enzyme cyclooxygenase (COX), which converts arachidonic acid into prostaglandins [153]. Although AD has no cure, several research lines have proposed the use of NSAIDs to alleviate symptoms [154]. NSAIDs have been used since the 2000s, and their use for AD has been tested in animal models and clinical trials [155–157]. Indeed, experiments in animal models of AD have shown that NSAIDs can be useful in this pathology. For example, in transgenic mice overexpressing APP, oral administration of ibuprofen, a nonspecific inhibitor of COX, at the onset of amyloid plaque formation decreased glial activation and plaque density [158, 159]. In another experiment, rats injected with Aβ in the dentate gyrus, treatment with indomethacin attenuated microglial activation, restored long-term enhancement of the hippocampus and prevented deficits in working memory. Moreover, in mice injected intracerebroventricularly with Aβ, increased COX-2 levels and memory impairment was induced [160]. Importantly, these alterations were attenuated by pretreatment with NS398, a selective COX-2 inhibitor. Additional reports have shown that ibuprofen and naproxen treatment in transgenic mouse models of AD were able to block microglial alterations without affecting APP processing [161]. There have also been studies in human cell cultures that have sparked hope regarding the treatment of AD with NSAIDs. For example, in an experiment using human neuroglioma cells overexpressing APP695NL, researchers observed that multiple NSAIDs such as sulindac, ibuprofen, and diclofenac selectively reduced Aβ42 [162]. Long-term placebo-controlled clinical trials have also demonstrated the usefulness of NSAIDs in AD. Among them is a study that evaluated the protective effects of naproxen, a nonselective COX inhibitor, and celecoxib, a selective COX-2 inhibitor, against AD in cognitively normal individuals over 70 years of age with a family history of AD [161]. Interestingly, subjects previously exposed to naproxen were 67% less likely to develop AD than control subjects previously exposed to placebo. However, it is important to note that these beneficial effects were observed only in people who had completely normal brains at the start of the study and not in people with existing brain disease (even if asymptomatic) at the start of the trial. It is worth noting that some of these people even worsened after NSAID administration [161, 163, 164]. Therefore, it can be suggested that chronic use of NSAIDs may be beneficial only in the early stages of the AD process, coinciding with the initial deposition of Aβ, the activation of microglia and the subsequent release of proinflammatory mediators. When the Aβ deposition process has already begun, NSAIDs are useless and can even be harmful, as they inhibit microglial inflammation; such inflammation, despite having harmful effects, mediates the elimination of Aβ [161]. It has been observed in other clinical trials that the protective effects of NSAIDs are superior when the duration of administration is longer than 12 months [161]. In addition, it has been suggested that the treatment time required to obtain the full benefit is two years [165].

Among the best-characterized reasons why NSAIDs can help alleviate AD symptoms are through the inhibition of COX. Research involving several animal models of AD has shown altered COX-2 brain expression, together with reactive gliosis, and behavioral dysfunction [160]. The increase in COX-2 may arise from microglial activation directly or indirectly caused by Aβ [166]. Indeed, postmortem AD brains show elevated levels of COX-1 and COX-2, compared to control brains [167]. COX-2 expression also increases after microglial activation. In fact, transgenic mice that overexpress COX-2 develop an age-dependent deficit in spatial memory at 12 and 20 months that is accompanied by apoptosis of neurons and astrocytes [160]. Interestingly, COX-2 is expressed in high concentrations in degenerative cells of the brain, thus inhibiting COX may reduce the risk of developing AD [168]. One study measured LPS-stimulated inhibition of plasma prostaglandin E2 (PGE2), an index of COX-2 activity ex vivo in volunteers who received placebo or various doses of celecoxib, a selective COX-2 inhibitor [169]. The results indicated that the COX-2 activity was significantly lower in volunteers who received different doses of celecoxib than in volunteers who received a placebo. Another reason why NSAIDs are associated with delayed AD symptoms is their effect on prostaglandins. Studies on the inflammatory mechanisms of AD have revealed that prostaglandins released during the inflammatory reaction have a degenerative effect [170]. In addition, prostaglandins can cause Aβ levels to rise. The last reason is not directly related to the anti-inflammatory capacity of NSAIDs but rather is related to the fact that these drugs reduce Aβ and tau levels. Some NSAIDs, such as indomethacin and ibuprofen, among others, have been reported to reduce the production of amyloid Aβ42 independently of COX inhibition [165]. Other hypotheses state that NSAIDs can interact directly with Aβ, thus preventing its accumulation. For example, ibuprofen and indomethacin have been shown to decrease the production of Aβ42 in vitro and in AD transgenic mice [161].

## Animal models for the study of neuroinflammation in AD

The relationship between inflammation and AD discussed thus far was discovered through studies on animal models of AD. It is also very important to acquire knowledge about possible treatments or risk factors for AD, which can also be achieved through the use of animal models. Such research is essential because AD is a disease without a cure. However, animal models can be used to investigate new drugs or treatments that can improve symptoms or even cure the disease.

Several different types of animal models are used to study AD, such as transgenic mice or rat models. Transgenic animals reproduce aspects of AD, such as mice that overexpress APP or mice that express human tau isoforms with typical AD mutations [171]. An example of a transgenic mouse is the PDAPP mouse, which overexpresses APP under the control of the mini-promoter of PDGF. This mouse model shows many of the pathological characteristics of AD, such as extensive deposition of extracellular amyloid plaques, astrogliosis, and neuritic dystrophy [172, 173]. Another example is the transgenic mouse expressing a human isoform of tau that lacks the two amino-terminal inserts (since it has a P301L mutation) under the control of the murine PrP promoter [174]. These mice exhibit NFTs in the BM and brain, and in addition, 90% of these mice develop motor and behavioral disturbances by 10 months of age [173, 175]. A third animal model is *O. degus*, a rodent that develops AD pathology without genetic manipulation or intervention. At approximately 56 months of age, features of AD are observed, such as the expression of neuronal β-APP (β-APP695), a neural-specific isoform containing 695 amino acids, intracellular and extracellular deposition of Aβ, intracellular accumulation of tau protein and ubiquitin, a strong astrocytic response, and the appearance of markers of AD [176, 177]. In addition, AD can be studied by imitating the characteristics of AD in a non-transgenic way. Although these models are not considered AD models, they have been of great use in various findings. For example, injecting Aβ_1–42_ into mice induces neuronal death in the CA1 region of the hippocampus, activates astrocytes and microglia, induces the expression of nitric oxide synthase, and triggers memory loss, among other AD features [178–180].

Studies on animals have contributed to some major therapeutic advances. For example, experiments to demonstrate the effects of ANDRO on AD have been performed in *O. degus*. Indeed, one study analyzed the expression of the neuroinflammatory markers GFAP, IL-6, and COX-2. Higher levels of GFAP and IL-6 were measured in the hippocampi of old animals than in young animals. The authors also observed approximately 30% lower levels of GFAP and approximately 40% lower levels of IL-6 in the hippocampi of 56-month-old animals treated with ANDRO (injected intraperitoneally) than in those of control animals (injected with a vehicle) of the same age or in those of 12-month-old control animals. Therefore, evidence suggests that ANDRO treatment can significantly decrease neuroinflammation [7]. In another study, rats were injected intraventricularly with Aβ_1–42_ to emulate the presence of Aβ in AD. In that study, the authors studied the effects of GsRb1, an anti-inflammatory component of *Panax ginseng* and one of the most commonly used medicinal herbs in Asian and Western countries [181], on behavior and the levels of inflammatory mediators. After 2 weeks of Aβ_1–42_ injection their results revealed that the model rats (injected with Aβ_1–42_ and not treated with GsRb1) showed a significant increase in the time taken to find the hidden platform. No loss of locomotor performance was observed, suggesting that the increase in time was due to a slowed learning process. After 4 weeks of treatment with GsRb1, learning ability was restored in the treated group, as indicated by a decrease in the platform encounter time compared to that of the model group. Moreover, the number of COX-2-immunopositive cells within the hippocampus was significantly greater in the model rats than in the treated rats, indicating that GsRb1 decreased COX-2 expression and thus regulated behavior. Similar results were obtained for IkB-α, and for nNOS: the immunopositive cells were greater in number in the treated rats than in the model rats. Through the use of the Aβ_1-42_ injection model, the authors concluded that GsRb1 reversed changes in various markers of neuroinflammation in the hippocampus, suggesting that this anti-inflammatory agent can be used to develop antiaging drugs [8].

Finally, we will discuss a study in which the authors proposed that certain anti-Aβ antibodies exert their effects through an antibody-mediated microglial activation [9]. In that study, the researchers administered an anti-Aβ antibody (m3D6) that binds aggregated Aβ in a PDAPP transgenic mouse model, which was further engineered to contain green fluorescent protein (GFP)-expressing fluorescent microglia. Interestingly, animals injected with m3D6 had significantly more microglial cells and had almost twice the number of processes protruding from their cell bodies [9].

## Obesity and AD

**Fig. 3:**
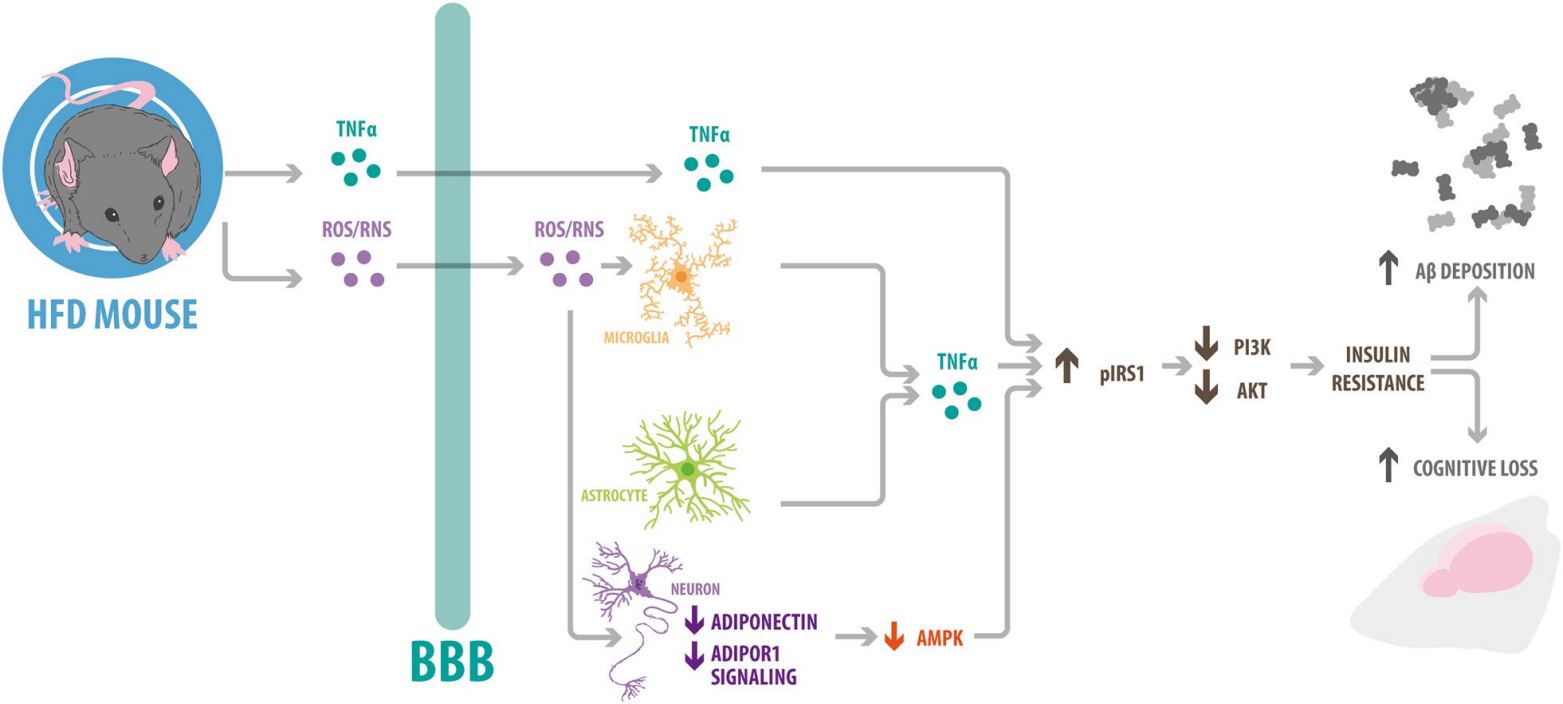
Brain inflammation produced in obesity. In HFD-fed mice, peripheral proinflammatory cytokines such as TNF-α and reactive species such as ROS and RNS are present. Both can cross the BBB to reach the brain. ROS and RNS activate microglia and astrocytes, stimulating them to produce more TNF-α and decreasing AdipoR1 signaling (which is why the levels of AMPK, which is part of this pathway, decrease). TNF-α (which enters the brain from the periphery and is produced by astrocytes and microglia when they are stimulated by ROS and RNS from the periphery), together with the decrease in AMPK, ultimately decrease IRS-1 signaling (IRS-1 is phosphorylated at serine instead of tyrosine, thus decreasing pPI3K and AKT levels). This causes insulin resistance, which is proposed to be the cause of increased Aβ deposition and cognitive loss

As discussed throughout this review, inflammation plays an important role in the pathogenesis of AD. Therefore, pathologies related to inflammation, such as stroke, traumatic brain injury, type 2 diabetes, metabolic syndrome, infection, sepsis, and obesity, are risk factors for AD [182–186]. Most cases of AD, approximately 95%, correspond to sporadic and nonfamilial AD, and thus it is important to understand the risk factors associated with this disease to help prevent it [187]. Among these factors is obesity, which has been proposed to influence AD progression [188]. According to the WHO, in 2016, approximately 40% of adults over 18 were overweight, and 13% were obese, while more than 340 million children and adolescents were overweight or obese [189]. Importantly, obesity has been shown to induce a state of inflammation [190]. For example, in one study, researchers analyzed the levels of the proinflammatory cytokine TNF-α in blood samples of obese and non-obese people ranging from 20 to 60 years old. Their findings showed that the TNF-α serum levels were significantly higher in obese individuals than in non-obese individuals [190]. In a different study, Hahm and colleagues evaluated the effect of a high-fat diet (HFD) on Aβ deposition and neuroinflammation [191]. Interestingly, their findings revealed that the HFD group exhibited a significant increase of approximately twofold in oxidative stress, measured by ROS and lipid peroxidation. This increase in oxidative stress also led to higher insulin resistance by impairing the adiponectin receptor 1 (AdipoR1) mediated AMPK-activated protein kinase (AMPK) signaling, which can exacerbate the pathogenesis of AD. Similarly, the suppression of AdipoR1 also resulted in increased oxidative stress and increased amyloidogenic pathway. Finally, the researchers observed that HFD feeding increased the levels of Aβ by more than 50% in the cortex and hippocampus (Fig. 3). Together, these results suggest that obesity, through inflammation, influences AD pathogenesis. Moreover, decreasing obesity, eating a healthy diet, and performing physical activity could be useful habits to prevent AD [192, 193].

## Conclusion

The contributions of various transgenic and non-transgenic animal models and inflammatory markers have made it possible to study the relationship between AD and inflammation, a well-known process characteristic of the pathogenesis of AD. Microglia and astrocytes, two main cell types in the CNS, play a critical role in the inflammatory process. These cells release proinflammatory cytokines when they are stimulated by insoluble aggregates such as Aβ and tau. In turn, this response induces synaptic loss and neurodegeneration and further increases Aβ and tau levels, triggering a vicious cycle. Due to their role in inflammation, several conditions, such as obesity, have been implicated as risk factors for AD. Importantly, potential AD treatments that restore the inflammatory phenotype, including the use of NSAIDs, are widely studied and developed. Indeed, clinical, and preclinical trials have already been carried out for this treatment with positive results. Thus, exploring these avenues could modify AD progression and pathology, improving the quality of life of many older adults and their families.

## Data Availability

Not applicable.

## Abbreviations

AD: Alzheimer’s disease
AMPK: AMP-activated protein kinase
Andro: Andrographolide
APOE: Apolipoprotein E
APP: Amyloid precursor protein
BBB: Blood–brain barrier
BM: Bone marrow
CNS: Central nervous system
EVs: Extracellular vesicles
GFAP: Glial fibrillar acidic protein
GMF: Glial maturation factor
GsRb1: Ginsenoside Rb1
IL-1β: Interleukin 1β
IL-10: Interleukin 10
IRS-1: Insulin receptor substrate 1
LRRs: Leucine-rich repeats
MHC: Major histocompatibility complex
NFTs: Neurofibrillary tangles
NF-κB: Nuclear factor kappa-activated light chain B
iNOS: Nitric oxide synthase
NSAIDs: Nonsteroidal anti-inflammatory drugs
PAR-2: Protease-activated receptor 2
PBMC: Peripheral blood mononuclear cell
PDAPP: Human amyloid precursor protein
PDGF: Platelet-derived growth factor
PGE2: Plasma prostaglandin E2
PPARγ: Peroxisome proliferator-activated receptor γ
ROS: Reactive oxygen species
RNS: Reactive nitrogen species
STAT: Signal transducer and activator of transcription
TNF-α: Tumor necrosis factor α
TGF-β: Transforming growth factor beta
TLRs: Toll-like receptors
WHO: World Health Organization
